# The Current State of Knowledge on *Salvia hispanica* and *Salviae hispanicae semen* (Chia Seeds)

**DOI:** 10.3390/molecules27041207

**Published:** 2022-02-11

**Authors:** Sara Motyka, Katarzyna Koc, Halina Ekiert, Eliza Blicharska, Katarzyna Czarnek, Agnieszka Szopa

**Affiliations:** 1 Chair and Department of Pharmaceutical Botany, Jagiellonian University Medical College, ul. Medyczna 9, 30-688 Kraków, Poland; sara.motyka@doctoral.uj.edu.pl (S.M.); koc.kat.17@gmail.com (K.K.); halina.ekiert@uj.edu.pl (H.E.); 2 Department of Analytical Chemistry, Medical University of Lublin, Chodźki 4a St., 20-093 Lublin, Poland; 3 Institute of Health Sciences, Faculty of Science and Health Sciences in Lublin, The John Paul II Catholic University of Lublin, ul. Konstantynów 1 H, 20-708 Lublin, Poland; kczarnek@kul.pl

**Keywords:** *Salvia hispanica*, chia, chia seeds, essential fatty acids, essential amino acids, biological activity, medicinal uses, cosmetological application, functional food

## Abstract

Chia seeds (*Salviae hispanicae semen*) are obtained from *Salvia hispanica* L. This raw material is distinguished by its rich chemical composition and valuable nutritional properties. It is currently referred to as “health food”. The purpose of the present work was to perform a literature review on *S. hispanica* and chia seeds, focusing on their chemical composition, biological properties, dietary importance, and medicinal uses. The valuable biological properties of chia seeds are related to their rich chemical composition, with particularly high content of polyunsaturated fatty acids, essential amino acids, polyphenols, as well as vitamins and bioelements. The available scientific literature indicates the cardioprotective, hypotensive, antidiabetic, and antiatherosclerotic effects of this raw material. In addition, studies based on in vitro assays and animal and human models have proven that chia seeds are characterized by neuroprotective, hepatoprotective, anti-inflammatory, and antioxidant properties. These properties indicate a valuable role of chia in the prevention of civilization diseases. Chia seeds are increasingly popular in functional food and cosmetic and pharmaceutical industries. That is attributed not only to their desirable chemical composition and biological activity but also to their high availability. Nevertheless, *S. hispanica* is also the object of specific biotechnological studies aimed at elaboration of micropropagation protocols of this plant species.

## 1. Introduction

*Salvia hispanica* L. is a herbaceous plant from the genus *Salvia* (sage) and *Lamiaceae* family (*Labiatae*). The genus *Salvia* is comprised of about 900 species which occur in almost all parts of the world (North, Central, and South America, South Africa, Southeast Asia, and Europe) [1,2,3,4,5,6,7,8,9]. The current European Pharmacopoeia (10th edition) [10] contains monographs of raw materials obtained from different *Salvia* spp., namely, *S. officinalis* (medical sage), *S. lavandulifolia* (lavender sage), *S. triloba* (Greek sage), *S. miltiorrhiza* (red root sage or red sage), and *S. sclarea* (clary sage). These monographs indicate the valuable healing properties of *Salvia* ssp. [11]. Among the mentioned *Salvia* spp., *S. officinalis* has long been used in European medicine [12]. The other species (except *S. miltiorrhiza*) were introduced to official European therapy in 2008 and were included in the 6th edition of the European Pharmacopoeia. The pharmacopoeial raw materials obtained from *S. officinalis* and *S. triloba* are leaves (*folium*). They are a source of tannins, which have an astringent effect, and also contain essential oil (Latin: *Salviae aetheroleum*), which has an anti-inflammatory effect [12,13]. Essential oils extracted from the leaves of *S. lavandulifolia* and *S. sclarea* also have anti-inflammatory and disinfectant properties [14,15]. A unique species of *Salvia* is *S. miltiorrhiza*, which was first introduced to official European healthcare in 2013 and included in the 7th edition of the European Pharmacopoeia. The raw material obtained from this species is the root and rhizome (*radix et rhizoma*) which is rich in diterpenes (tanshinones) and salvianolic acid B. It is mainly used to treat cardiological disorders, and also has antimicrobial effects [16].

**Figure 1:**
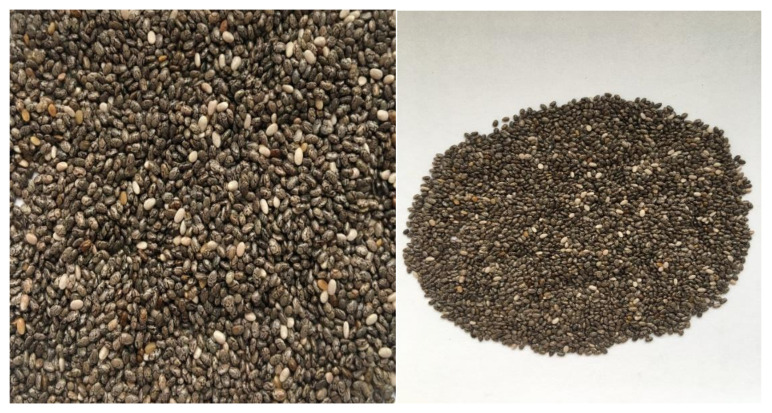
Morphological appearance of *S. hispanica* seeds.

Among *Salvia* spp., currently one of the most valuable and widely used is undoubtedly *S. hispanica* L. (chia). The raw material obtained from this species is seed (*Salviae hispanicae semen*) (Figure 1). The species name “*hispanica*” incorrectly refers to the Spanish origin of the plant. This error resulted from the mistake by Carl Linnaeus who confused *S. hispanica* with *S. lavandulifolia* [17], which is known in English as “Spanish sage” and is native to Spain (and southern France) [17,18].

*S. hispanica*, according to historic sources, was already known to the Aztecs, who used chia seeds in food and as an ingredient in many herbal mixtures, although no specific healing properties were assigned to them [3]. Oil obtained from chia seeds was also used to produce cosmetics, as a solvent for painting, and in religious rituals [19]. The term “chia” is derived from the Spanish word “chian,” which means “oily” [2,4,9,20,21,22,23]. It is also thought that the word “chia” originated from the word “chihaan,” from the Mayan language, meaning “strong” or “strengthening” [3]. The Plant List database provides the following Latin synonyms for *S. hispanica*: *Kiosmina hispanica* (L.) Raf., *Salvia chia* Colla, *Salvia chia* Sessé & Moc., *Salvia hispanica* var. *chionocalyx* Fernald, *Salvia hispanica* var. *intonsa* Fernald, *Salvia mexicana*, *Salvia neohispanica* Briq., *Salvia prysmatica* Cav., *Salvia schiedeana* Stapf., and *Salvia tetragona* Moench [24]. The most common and colloquial name of *S. hispanica* is chia. Other names, such as “chan,” “ican,” and “cueruni”, are used in the areas of natural habitats of the plant [3]. In Portuguese and Spanish, *S. hispanica* is also known as chia [25]. The species is also called chian, salvia chia, salvia chian, salba, and black chia [17].

The species *S. hispanica* does not have its monograph in the European Pharmacopoeia, nor in any national pharmacopoeias of the European Union and other European countries [26,27]. In 2019, the seeds of *S. hispanica* were approved by the European Food Safety Authority (EFSA) as a “novel food for extended uses, which allowed the inclusion of chia seeds in a variety of foods” [28,29]. Moreover, chia seeds have been added to the list of cosmetic raw materials in the CosIng database (Cosmetic Ingredient Database) which was developed by the European Commission [30].

Chia seeds, as a valuable and increasingly popular component of “healthy food”, have been the focus of previously published review articles [6,9,31,32,33,34,35,36,37,38,39]. The authors emphasized the importance of chia seeds as a valuable new food resource with high health and antioxidant potential.

Their unique chemical composition and high nutritional value make chia seeds a very valuable raw material used on an industrial scale. In order to preserve all essential nutrients at high levels, the latest techniques used in nanotechnology are applicable. Nanotechnology promotes improved quality, safety, bioavailability, better controlled release, more accurate targeting, and enhances applicability. In the case of seeds obtained from oil plant species, nanoemulsions derived from seed oils are widely used to create nanocarriers that transport valuable biologically active compounds. The small particle size determines high bioavailability and gravitational stability. Encapsulation of chia seed oil results in increased bioavailability of the encapsulated active ingredient, protection from adverse effects, both natural and processed, such as chemical action, enzymatic action, and physical instability observed during processing. Encapsulation represents a very important and innovative way to improve biological performance and enhance health outcomes by controlling the delivery of active ingredients and preventing the appearance of side effects. Numerous scientific studies confirm the use of the lipid fraction of chia seeds to create nanoemulsions and nanoliposomes, which have a wide range of health-promoting therapeutic applications [39,40,41,42,43,44,45,46].

The aim of our publication is to bring together all recent scientific reports and to present, in their context, chia seeds as a raw material with a strong, well-established health-promoting position. Moreover, the paper presents information on the botanical characteristics and occurrence of *S. hispanica*. It describes in detail the latest scientific research on the biological activities of chia seeds in the context of their use in the treatment and prevention of civilization diseases. The paper is the latest compendium of knowledge on *S. hispanica* species as well as the chia seeds (*Salviae hispanicae semen*).

## 2. Morphology, Natural Habitats, and Cultivation

*Salvia hispanica* is an annual plant. It grows up to a height of 1 m. The leaves of this plant are elongated and serrated, arranged opposite, and measure 3–5 cm wide and 4–8 cm long. The flowers are bisexual, white or purple in color, arranged in whorls, have a labial structure typical of the Lamiaceae family, and a dimension of 3–4 mm. The seeds are oval in shape and measure 1–2 mm in length. They can appear uniform or as mixed and speckled (Figure 1) [7,8,9,34,47,48]. Their color can vary from white through gray to black. The white color of the seeds is a recessive trait, which is determined by a single gene and “exposed” by some chia growers. An example of the breeding variety with white seeds is *S. hispanica* cv. Salba. Agrisalba SA (Lima, Peru) declares that compared to darker chia seeds, white seeds have a higher and more stable content of omega-3 fatty acids, especially α-linolenic acid (ALA) [17,49].

Some of the features selected for cultivation and that distinguish the *Salvia* cultivars from wild *S. hispanica* are as follows: size, color, and surface area of seeds; apical dominance; increased number of branches; increased inflorescence length and calyx height; and shortened pubescence. The most important feature selected for artificial crop cultivation is closed cups, which prevent seed dispersal and eliminate domesticated varieties outside of breeding [3].

The species *S. hispanica* is indigenous to southern Mexico and Guatemala. It can be found in tropical and subtropical climatic zones, and its natural habitats are mostly mountainous areas. The plant grows well in sandy loam, loam, and loam soils with good drainage. It thrives mainly in acidic soils and grows best at a pH of 6.5–8.5. Temperatures between 11 and 36 °C are ideal for seed growth. Under appropriate agroclimatic conditions, the plant can produce about 600 kg seeds/acre. Mexico is currently the world’s largest producer of chia seeds. On an industrial scale, *S. hispanica* is cultivated almost all over the world, including Central and North America (Guatemala, Honduras, Mexico, Nicaragua, Panama, and USA), South America (Argentina, Bolivia, Brazil, Ecuador, Colombia, Paraguay, and Peru), Europe, and Australia. Seeds of *S. hispanica* are grown in greenhouses in Europe. According to data from 2014, the global artificial cultivation of *S. hispanica* accounted for 370,000 ha in 33 countries [34,48,50,51,52].

## 3. Bioactive Components of *S. hispanica* Seeds

Seeds are the major raw material obtained from *S. hispanica* on an industrial scale. Chia seeds are considered to have high nutritional value mainly due to their high content of polyunsaturated fatty acids (PUFA) and dietary fiber. In addition, the seeds contain wholesome protein and several phenolic compounds, macro- and microelements, and vitamins. Chia seed sprouts are also characterized by desirable chemical composition and high nutritional value [7,8,9,47,53].

Based on research, it has been proven that the chemical composition of chia seeds can change under the influence of various external factors. As shown by scientific studies, the content of individual nutrients can vary depending on the origin of the plant, harvesting time, storage, drying, area where the plant is grown, terrain, germination process, starting date, availability of essential nutrients, and rainfall (amount, frequency, intensity) [27,32,47,54,55].

Oil obtained from the seeds of *S. hispanica* accounts for 30–33% of fatty acids [54], of which 80% are essential fatty acids (EFAs), especially PUFA (Figure S1, Supplementary Materials; Table 1). The dominant fatty acids in the oil are ALA, which is an omega-3 fatty acid constituting about 60% of the fatty acid pool, and linoleic acid (LA), which is an omega-6 fatty acid constituting about 20% of oil. The ratio of omega-6 to omega-3 fatty acids in chia seeds is very favorable (around 0.3–0.35) [2,7,9,20,39,54,55,56,57,58,59,60,61]. Monounsaturated fatty acids, which belong to the group of omega-9 fatty acids, constitute about 10% of the fatty acid pool in chia seeds, with oleic acid being dominant (Table 1). Saturated fatty acids constitute the remaining 10% of the fatty acid pool, and the dominant ones are palmitic and stearic acid (Figure S1) [62]. Compared to raw materials from other plant species, chia seeds are the richest source of ALA. Unfortunately, the high content of PUFA (80%) in chia seeds makes them susceptible to oxidation processes, which decreases the nutritional value of the seeds [39].

Carbohydrates account for 26–41% of the weight of *S. hispanica* seeds [54]. The seeds contain about 30–34% of dietary fiber (made of plant carbohydrate polymers, both oligosaccharides and polysaccharides, such as cellulose, hemicelluloses, pectic substances, and gums, which may be associated with lignin and other noncarbohydrate components) [65]. The insoluble fiber fraction of chia seeds is about 85–93%, and the soluble fiber fraction is about 7–15%. Chia seeds have a higher amount of dietary fiber compared to dried fruits of other plant species, cereals, and nuts, which are considered rich sources of dietary fiber [66,67].

Protein makes up 15–25% of the weight of *S. hispanica* seeds. The main storage protein found in chia seeds is globulin, which makes up 52% of the total protein content. Albumins (17%), glutelins (14%), and prolamins (12%) are the other proteins found in lower abundance. Chia protein has a good digestibility (78.9%), similar to that of casein (88.6%), higher than maize (66.6%), rice (59.4%), and wheat (52.7%) proteins, but less than amaranth (90%). In comparison to other types of cereals, such as corn seeds (9.4%), rice (6.5%), quinoa (14.1%), and wheat (12.6%), chia seeds have higher protein content. The protein fraction of chia seeds includes a set of exogenous amino acids [9,32,34,47,60,68,69,70], namely, arginine (Arg), phenylalanine (Phe), histidine (His), isoleucine (Ileu), leucine (Leu), lysine (Lys), methionine (Met), threonine (Thr), tryptophan (Trp), and valine (Val). Endogenous amino acids found in chia seeds include: alanine (Ala), cysteine (Cys), serine (Ser), tyrosine (Tyr), aspartic acid (Asp), and glutamic acid (Glu) (Figure S2, Supplementary Materials). Among the amino acids found in *S. hispanica* seeds, Glu is dominant (3.50 g/100 g dry seeds). Arg and Asp are also found in a high amounts (2.14 and 1.69 g/100 g dry seeds, respectively), while others constitute less than 1 g/100 g dry seeds [71].

The seeds of *S. hispanica* contain polyphenol compounds that exist either in a free form or bound by glycosidic bonds with sugars. The following polyphenols have been identified in chia seeds: phenolic acids (gallic acid, caffeic acid, ferulic acid, *p*-coumaric acid), depsides (chlorogenic acid, rosmarinic acid) (Figure S3, Supplementary Materials), flavonoids (apigenin, kaempferol, quercetin, myricetin, rutoside), isoflavones (daidzein, glycitin, genistein, genistin), and catechin derivatives (including epicatechin) (Figure S4, Supplementary Materials).

Rosmarinic acid is the dominant phenolic acid with the highest concentration (0.927 mg/g), followed by protocatechuic acid (0.747 mg/g), caffeic acid (0.027 mg/g), and gallic acid (0.012 mg/g) (Table 2). Tannins and phytates are also found but in negligible amounts. Carotenoids (carotenes and xanthophylls) are present at an amount of about 0.5 μg/g [5,34,55].

Chia seeds also contain the following sterols in small amounts: campesterol (472 mg/kg), stigmasterol (1248 mg/kg), β-sitosterol (2057 mg/kg), and ∆5-avenasterol (355 mg/kg) [34,59].

Furthermore, *S. hispanica* seeds contain a significant amount of valuable bioelements. These include macroelements, such as phosphorus (P), iron (Fe), manganese (Mn), calcium (Ca), potassium (K), magnesium (Mg), sodium (Na), or sulfur (S), and microelements, such as zinc (Zn), copper (Cu), molybdenum (Mo), and selenium (Se) (Table 3). Compared to other cereals, such as wheat, rice, and maize, the content of P, Ca, K, and Mg in chia seeds is higher. In addition, *S. hispanica* seeds are an especially rich source of Ca (560–800 mg/100 g dry seeds) and Mg (325–460 mg/100 g dry seeds) [47].

The seeds of *S. hispanica* contain vitamins, namely, B-complex, C, A, and E (Table 4). The content of individual vitamins in 100 g of dry chia seeds is: 8.83 mg of vitamin B3, 1.6 mg of vitamin C, 0.62 mg of vitamin B1, 0.50 mg of vitamin E, 0.17 mg of vitamin B2, and 54 µg of vitamin A [7,73,77]. The share of individual tocopherols in the content of vitamin is as follows: α-tocopherol—8 mg/kg, γ-tocopherol—422 mg/kg, and δ-tocopherol—15 mg/kg [59].

## 4. Chemical Composition of *S. hispanica* Leaves

The literature indicates that the majority of studies on *S. hispanica* have focused on the raw material, i.e., seeds, while only a few have analyzed the chemical composition of leaves and other parts of the chia plant. Research on chia leaves looks at their antioxidant composition and antimicrobial properties [75,79].

In 2015, Amato et al. demonstrated the presence of flavonoids and derivatives of hydroxycinnamic acid in *S. hispanica* leaves. The flavonoid content included flavones (apigenin, luteolin, orientin, myricetin, and vitexin) as well as flavonols (derivatives of quercetin and kaempferol). In addition, researchers have isolated and identified two compounds—acetyl derivatives of vitexin and orientin. Among the derivatives of hydroxycinnamic acid, the presence of caffeic acid, *p*-coumaric acid, and rosmarinic acid was confirmed in the leaves of *S. hispanica* [75]. This study aimed to evaluate the antioxidant capacity of chia leaf extracts for both white and black seed phenotypes. Three methods were applied for measurements—oxygen radical absorbance capacity (ORAC), 2,2′-diphenyl-1-picrylhydrazyl (DPPH), and ORAC-Fluoresceine (ORAC-FL). In addition, cellular antioxidant activity (CAA) assays were performed on methanolic and ethanolic extracts of chia seeds. According to the ORAC-FL index values, the extracts prepared with less polar solvents (dichloromethane and hexane) had higher antioxidant capacity. Leaf extracts with black seed phenotype extracted with ethanol solvent had the highest ORAC-FL index (2.72 ± 0.09). The study documented that the ethanolic leaf extract of black phenotype showed the best quality and antioxidant capacity, both considering the stoichiometry and reactivity of antioxidants in the tested extracts. Moreover, the ethanolic extracts were found to have the highest concentration of rosmarinic acid. In extracts with more polar solvents (ethanol and ethyl acetate), the highest amounts of bioactive compounds were identified, mainly: caffeic acid, rosmarinic acid, protocatechuic acid, *p*-coumaric acid, coumaric acid-*O*-hexose, kaempferol, and genistein. In addition, the F isomer of salvianolic acid and dimethylquercetin were found only in ethyl acetate and dichloromethane extracts. Caffeic acid as well as rosmarinic acid have been confirmed in the *S. hispanica* leaf extracts before [79].

## 5. Biological Activities of *S. hispanica* Seeds Confirmed by Scientific Research

### 5.1. Hypoglycemic, Hypotensive, Hypolipemic, Hepatoprotective, and Fat-Reducing Effects

#### 5.1.1. Animal Model Studies

Researchers from the University of Campinas (Campinas, São Paulo, Brazil) assessed the impact of consumed chia seeds on the metabolic rates of selected carbohydrates. They found that rats fed with a high-fat, fructose-rich diet, in which soybean oil was replaced by chia seed oil, or a high-fat, high-fructose diet containing 13.3% of chia seeds showed greater tolerance to both high glucose and high insulin levels compared to rats fed with a standard high-fat, high-fructose diet. This effect of greater glucose and insulin tolerance was observed in both short- (6-week) and long-term (12-week) dietary interventions. In addition, the group of animals consuming *S. hispanica* seeds showed a decrease in blood concentrations of non-esterified fatty acids. Furthermore, these animals showed a decrease in the level of hepatocyte damage markers, namely, alanine transaminase (ALT) and aspartate transaminase (AST), both of which tend to be increased by high-fat and high-fructose diets [80].

A study conducted at the University of Litoral (Santa Fe, Argentina) demonstrated the beneficial effects of *S. hispanica* seeds on the lipid profile in rats. Rats fed with a high-sucrose diet containing 2.6% of chia seeds had lower concentrations of triglycerides (TGs), non-esterified fatty acids, and total cholesterol (TC) compared to rats fed with the same diet lacking chia seeds. The addition of chia seeds to the diet had a positive effect on the glucose metabolic pathway and reduced collagen deposition in the heart of dyslipidemic, insulin-resistant rats fed with sucrose-rich diet. The levels of blood glucose were not altered in the studied rats. Additionally, a reduction in the thickness of visceral adipose tissue was observed in rats consuming chia seeds [81,82]. 

Subsequent studies at the same center also confirmed the beneficial effects of *S. hispanica* seeds on lipid profile. Replacing corn oil with chia seeds as a fat source in a sugar-rich diet prevented (in the group of rats in which the change was made from the beginning of the experiment) or improved and normalized (in the group of rats in which the change was made after 3 months) dyslipidemia, liver TG levels, and activities of fatty acid synthase, acetyl-CoA carboxylase, glucose-6-phosphate dehydrogenase, fatty acid oxidase, and carnitine palmitoyltransferase [64].

A team from the Federal University of Viçosa (Viçosa, Brazil) demonstrated that rats receiving a diet containing *S. hispanica* seeds or seed flour had lower blood levels of TGs, TC, low-density lipoprotein (LDL), and very-low-density lipoprotein (VLDL) compared to control rats receiving casein and non-protein diets. In addition, these animals showed increased levels of high-density lipoprotein (HDL) compared to controls. The study also proved that *S. hispanica* seeds exhibited hypoglycemic effects [83].

Fonte-Faria et al. from Rio de Janeiro State University (Brazil) examined the effects of diet supplemented with *S. hispanica* seed oil on the body weight composition and insulin signaling in the skeletal muscles of obese mice. Mice that received a chia seed oil-supplemented diet showed reduced fat accumulation and increased lean mass. In addition, these animals showed better blood glucose levels and better insulin tolerance as well as increased levels of HDL [82].

A study by Marineli et al. aimed to evaluate the effect of *S. hispanica* seeds and seed oil in the diet on total plasma antioxidant potential (TAS) and liver in obese rats. It was found that among rats consuming chia seeds or seed oil, there was a statistically significant increase in the activity of plasma antioxidant enzymes, i.e., catalase (CAT) and glutathione peroxidase (GPx), as well as an increase in glutathione (GSH) concentration, compared to the control group (receiving a standard diet rich in fat and fructose). In rat livers, glutathione reductase (GRd) activity was increased while CAT and GPx activities were unchanged. In addition, blood concentrations of lipid peroxidation biomarkers were decreased in animals receiving seed or seed oil diets: 8-isoprostane and TBARS (thiobarbituric acid reactive substances formed as a by-product of lipid peroxidation) compared to the control group. The assayed antioxidant capacity in plasma and liver was higher in rats receiving *S. hispanica* seeds and seed oil by 35% and 47%, respectively, compared to the control group [67].

#### 5.1.2. Clinical Studies

A study conducted at the Appalachian State University (Kannapolis, North Carolina, USA) assessed the effect of chia seed consumption on the blood levels of selected fatty acids in postmenopausal women. The results showed that daily intake of 25 g of chia seeds for 7 weeks resulted in an increase in the blood levels of ALA (by 138%) and eicosapentaenoic acid (by 30%) in the studied women. No differences were observed in the levels of docosapentaenoic and docosahexaenoic acids [74].

Researchers from the Federal University of Paraíba (João Pessoa, Brazil) investigated the effects of dietary supplementation with *S. hispanica* seed flour on blood pressure and the related cardiometabolic factors in treated and untreated hypertensive people. Hypertensive patients were randomly assigned to one of the following groups: group treated with the drug, untreated group, and placebo group. All patients consumed 35 g of chia seed flour or placebo daily for 12 weeks. The subjects who were treated with the drug and consumed *S. hispanica* seed flour-supplemented diet showed a decrease in mean blood pressure from 111.3 to 100.1 mmHg. The subjects who were not treated but consuming the flour-supplemented diet showed a mean decrease in systolic blood pressure from 146.8 to 137.3 mmHg. The placebo group showed no changes in blood pressure [84].

A study conducted at the Clinical Nutrition and Risk Factor Modification Centre at St. Michael’s Hospital (Toronto, Canada) on patients with type 2 diabetes showed that daily intake of 15 g/1000 kcal of *S. hispanica* seeds for 12 weeks resulted in a statistically significant reduction in the level of high-sensitivity C-reactive protein (CRP, by 40%) as well as in the level of von Willebrand factor (by 21%), a component of blood involved in clotting. In the patients consuming *S. hispanica* seeds, systolic blood pressure decreased by an average of 6.3 mmHg. No statistically significant differences in blood glucose levels or blood lipid profile parameters (TC, LDL, HDL, TGs) were observed in the studied group of patients [85]. The results of the study indicated that the hydrolysates of chia proteins, which exhibit angiotensin-converting enzyme inhibitory activity, may be responsible for the hypotensive effect of chia seeds [86,87].

The abovementioned research center also conducted a study assessing the effect of dietary supplementation with chia seeds on the reduction of postprandial glycemia. The results confirmed that this effect was dependent on the dose of chia seeds. People who consumed chia seed-enriched bread had lower postprandial glycemia compared to those who consumed bread without chia seed supplementation. The lowest glycemic level was found in people who consumed bread with 24 g of chia seeds, and the highest level in people who consumed 7 g of seeds. The authors of the study suggested that the fiber contained in chia seeds may be responsible for their hypoglycemic effect [88]. Another study conducted at the same research center assessed the effect of dietary supplementation with *S. hispanica* seeds on body weight, visceral obesity, and obesity-related risk factors in overweight or obese type 2 diabetes patients who were on a calorie-restricted diet. The first group received 30 g/1000 kcal/d of *S. hispanica* seeds, and the second (control) group received 36 g/1000 kcal/d of wheat bran. The primary effect observed was weight change over 6 months, and the subsequent end-effects noted were changes in waist circumference, body composition, glycemic control, and levels of CRP and obesity-related satiety hormones. After 6 months, the group receiving *S. hispanica* seeds showed a greater weight loss (1.9 kg) compared to the control group (0.3 kg), as well as a greater loss of waist circumference (by 3.5 and 1.1 cm, respectively) [89].

The use of *S. hispanica* seeds may be effective in patients with non-alcoholic fatty liver disease (NAFLD). This is attributed to the omega-3 EFAs, dietary fiber, and polyphenols contained in chia seeds. The introduction of chia seeds in the diet may promote the reduction of intrahepatic fat in patients with NAFLD. In a study conducted by Medina-Urrutia et al. involving 25 patients with NAFLD and insulin resistance, dietary supplementation with ground chia seeds at a dose of 25 g/d for 8 weeks resulted in a reduction in body weight (median-.4%) and waist circumference. In addition, reductions in total cholesterol (from 4.8 to 4.6 mmol/L) and triglyceride levels (from 1.9 to 1.6 mmol/L) were observed in the intervention group [90].

### 5.2. Antioxidant and Neuroprotective Effect

Researchers from the State University of Maringá (Maringá, Brazil) have shown in vitro that the ingredients of *S. hispanica* seeds are capable of inactivating ABTS (2,2′-azino-bis-3-ethylbenzothiazoline-6-sulfonic acid), eliminating synthetic DPPH (2,2-diphenyl-1-picrylhydrazyl) radicals, and reducing iron (III) ions evaluated by FRAP (ferric reducing antioxidant power) test. The antioxidant capacity of the samples determined by ABTS, DPPH, and FRAP tests was 2.56, 1.72, and 2.86 mmol equivalent antioxidant capacity of the reference antioxidant Trolox/g sample, respectively [91].

Scientists at the National Autonomous University of Mexico (Mexico) also investigated the antioxidant activity of chia seed ingredients. They confirmed the antioxidant activity of chia seeds by the ABTS test, and through the inhibition of lipid peroxidation as well as β-carotene oxidation in the β-carotene and linoleic acid model system. The antioxidant capacity of *S. hispanica* seeds was found to be comparable to that of Trolox used as the reference substance. This study also confirmed the antioxidant activity of *S. hispanica* seed oil used in a model food emulsion of the water/oil type [66].

A study was conducted at the University of Campinas (Campinas, São Paulo, Brazil) to evaluate the effects of *S. hispanica* seed and seed oil added in the diet on the oxidative state of plasma and liver in obese rats. Rats consuming chia seed or oil showed significantly increased activity of antioxidant enzymes in plasma (i.e., catalase (CAT) and glutathione peroxidase (GPx)) as well as significantly increased concentration of glutathione (GSH), in comparison to the control group (receiving a standard high-fat and high-fructose diet). The activity of glutathione reductase (GRd) was found to be increased in the livers of experimental animals, while the activity of CAT and GPx remained unchanged. In addition, animals receiving seeds or seed oil in their diet showed decreased blood levels of lipid peroxidation biomarkers, namely, 8-isoprostane and TBARS (substances reacting with thiobarbituric acid, formed as a by-product of lipid peroxidation), compared to the control group. The experimental group also showed higher antioxidant capacity in plasma and liver (by 35% and 47%, respectively), compared to the control group [67].

A study by Leo et al. investigated the neuroprotective effects of *S. hispanica* peptide fractions. Using enzymatic hydrolysis of chia proteins, three peptide fractions (1, 1–3, and 3–5 kDa) were produced and examined for protective and anti-inflammatory effects on HMC3 microglia cells. The 1–3 kDa fraction of chia seeds showed the greatest neuroprotective effect in HMC3 cells. Its action resulted in a reduction in the levels of inflammatory mediators (TNF-α, IL-6, NO, and H_2_O_2_) and reactive oxygen species (ROS). The authors suggest that the use of bioactive peptides derived from chia seeds may have beneficial applications in the prevention of neurodegenerative diseases through their anti-inflammatory and antioxidant activities [92].

### 5.3. Action against Kidney Stones

Saleem et al. in their study used a rat model of urolithiasis that was induced by the application of ethylene glycol. Added with ammonium chloride, ethylene glycol administered for three days accelerated the induction of urolithiasis in albino rats. Thirty-six rats were divided into six groups, the first group was the control, the second group had ethylene glycol (0.75% v/v) administered, the third group received standard drug—Cystone (Himalaya Drug Company, India) (750 mg/kg b.w.), the remaining groups were administered methanolic extracts of chia seeds (100, 300, and 700 mg/kg b.w., orally). Reduced serum creatinine, urea, uric acid, urea nitrogen, total protein, and albumin levels were observed in the experimental group after administration of the chia seed extract compared to the reference control values. In the control group where ethylene glycol was administered, creatinine, uric acid, blood urea nitrogen, total protein, and albumin levels (0.84 md/dL, 4.56 md/dL 14.17 md/dL, 6.14 g/dL, and 3.74 g/dL) were elevated compared to normal control values (2.75 md/dL, 0.56 md/dL, 9.14 md/dL, 5.44 g/dL, and 3.06 g/dL). In addition, there was a reduction in urinary calcium, oxalate, and phosphate levels, indicating that the chia seed extract prevented urolithiasis formation. In vitro studies showed that chia seed extract prevented calcium oxalate stone formation by inhibiting the initial stages of CaOx crystal formation, including nucleation, aggregation, and growth phases. The authors suggested that flavonoids present in chia seeds are responsible for this activity, mainly quercetin, which through its antioxidant potential prevents the precipitation of kidney stones [36,93,94,95] (Table 5).

### 5.4. Improving the Function of the Digestive Tract

The use of soluble extracts from plants rich in dietary fiber, including chia seeds, may have a positive effect on gastrointestinal motility, improving absorption of vitamins and minerals [98,99,100,101,102]. Soluble extracts are obtained by isolating prebiotics from food matrices, which mostly consist of water-soluble dietary fiber. Studies show that the use of such extracts improves the absorption of nutrients by regulating the expression of specific proteins of the intestinal brush-border membrane, in addition to an increase in the surface area of the intestinal villi and increased mucilage production [103]. Administration of seed extracts causes increased fermentation processes in intestines, production of short-chain fatty acids by intestinal bacteria, which contributes to lowering of pH and creation of conditions unfavorable for pathogenic intestinal bacteria. When a soluble extract of plant origin is administered, there is an increase in the absorption of minerals, especially an increase in the bioavailability of zinc and iron is observed [104,105].

The study by Silva et al. was designed to evaluate the effect of intra-amniotic administration of soluble methanolic prebiotic chia extracts on Fe and Zn levels and on brush-border membrane functionality in vivo. Researchers demonstrated that the use of methanolic extract of chia seeds results in a beneficial improvement in intestinal function; including increased surface area, thickness, and width of intestinal villi, increased proliferation of enterocytes, and increased mucilage secretion through an increase in the number of goblet cells. The researchers also examined the effects of the extracts on changes in intestinal bacterial populations and intestinal morphology.

The levels of Fe, Zn, insoluble fiber fraction, and phytic acid content were higher in chia seeds compared to chia seed extract. However, the water-soluble fiber content was significantly higher in chia seed extract compared to chia seeds. In the groups treated with chia extract, the area, length, and width of intestinal villi, including cecal mass, were higher compared to the control group. The researchers concluded that soluble chia extracts had beneficial effects on intestinal microflora by affecting enterocyte proliferation and increasing the number of mucus-producing cells, this may increase the digestive and absorption capacity of the intestinal brush-border membrane (BBM). In addition, the use of chia extract resulted in an increase in cup cell diameter compared to the control group. Administration of soluble chia seed extracts enhanced the expression of proteins involved in zinc metabolism. In the group treated with chia extract (2.5%), the expression of DMT1 (divalent metal transporter 1) genes was lower compared to the control group. Moreover, other concentrations of methanolic chia extract did not affect DMT1 gene expression. DcytB (duodenal cytochrome b) and hepcidin expression were significantly increased in the groups treated with 1%, 2.5%, and 5% chia extract, which could potentially increase the efficiency of iron absorption. In addition, groups treated with 1%, 2.5%, and 5% chia extract had lower ferroportin gene expression compared to other groups. There was also a beneficial effect of the administration of soluble chia extracts (0.5%) on the composition of the intestinal microflora by increasing the number of beneficial intestinal bacteria of the genera *Bifidobacterium* and *Lactobacillus* in the cecum. Increased numbers of *Lactobacillus* and *Bifidobacterium* may also contribute to increased bioavailability of minerals, as these bacteria produce short-chain fatty acids (SCFAs) that lower intestinal pH and therefore may increase solubility and absorption of Fe and Zn. In this study, intra-water (in ovo) administration of soluble chia seed extracts was shown to benefit gastrointestinal function by increasing mineral bioavailability and improving intestinal morphology [56] (Table 5).

## 6. Application of *S. hispanica* in Cosmetology

CosIng (Cosmetic Ingredient Database), a database developed by the European Commission, contains information on cosmetic substances authorized by the European legislation for the production of cosmetics.

The CosIng database provides information on six raw materials obtained from *S. hispanica*: whole and powdered seeds, chia seed extract and oil, and *S. hispanica* herb extract and oil. It also has data on the activity profile of raw materials, including the peeling and softening of seeds, as well as moisturizing and nourishing effects of seed oil. In addition, *S. hispanica* oil and herb extract are used for flavoring and recommended for the production of perfumes (Table 6) [30].

The health-promoting effects of phenolic acids present in chia seeds, which have antioxidant and antimicrobial properties, are particularly exploited in cosmetology. Due to these properties, phenolic acids are used in cosmetology and dermatology. These compounds can be found in both generally available cosmetic preparations, as well as in professional preparations used for treatments in beauty salons. Treatments with these acids counteract photoaging of the skin and also show depigmentation properties by controlling the activity of tyrosinase, which maintains an even pigmentation of the skin. In addition, phenolic acids alleviate the symptoms of acne and atopic dermatitis [106]. Table 6 shows the main raw materials extracted from *S. hispanica* registered in the CosIng database along with their uses.

The raw materials obtained from *S. hispanica* are especially applied in the production of natural and vegan cosmetics that are currently widely available on the market. The most common ingredients used in these cosmetics are chia seed oil and extract. Chia seed oil is also sold alone as a cosmetic product. The cosmetics prepared using *S. hispanica* extract and seed oil include hand and face creams, face oils, masks, foundations, micellar lotions, body lotions, shower gels, soaps, deodorants, shampoos, and hair conditioners. In all these cosmetics, chia extract and seed oil serve as a nourishing and moisturizing agent [107,108]. In addition, oil-in-water emulsions containing chia seeds are widely used in cosmetology, where they show a moisturizing effect on the skin (Table 6). Researchers at Korea University, College of Medicine (Ansan, South Korea), evaluated the benefits of topical application of omega-3-rich foods to the skin. They tested an oil/water emulsion containing 4% *S. hispanica* seed oil on five healthy volunteers with symptoms of xerotic pruritus and five patients with pruritus caused by end-stage renal disease or diabetes. After eight weeks of use, all patients showed a significant improvement in skin hydration, as well as an improvement in the epidermal barrier function, as confirmed by reduced transepidermal water loss and increased skin hydration. Application of the topical preparation with chia seed oil also reduced itching in all patients, with no adverse effects [109].

## 7. Applications of Chia Seeds in the Food Industry

Chia seeds are widely used in food production, especially in the functional food, due to their high lipid content (30–33%), proteins (15–25%), carbohydrates (26–41%), vitamins, bioelements, and dietary fiber (18–30%) [33,47,54,55]. The caloric value of 100 g of dry seeds is 486 kcal [71]. In 2019, the EFSA confirmed that chia seeds are safe to use in food products [28,29]. It was also shown that chia seeds do not contain mycotoxins or harmful levels of heavy metals [28,110]. According to the Advisory Committee on Novel Foods and Processes in the UK, adults should consume on average 2.1 g of chia seeds per day, with a maximum of 12.9 g (approximately a spoonful). The average level of consumption for children aged 1.5–4.5 years should be 1.1 g/day (maximum 3.2 g/day), and for those aged 4.5–19 years, the daily intake should not exceed 4.3 g/day [58].

Chia seeds are used in the food industry in the form of whole seeds, ground seeds, seed flour, or seed oil [7,111]. They can be added to various food products, such as juices, yogurts, cakes, cookies, bread, pasta, ice cream, desserts, breakfast cereals, and even sausages and hams. Chia seed oil is also available on the market and is recommended as an additive for sandwiches, salads, cottage cheese, and spreads. The US dietary guidelines published in 2000 recommend that chia seeds can be consumed at an amount not exceeding 48 g/day [8]. In addition, a new regulation was implemented in 2017 stating the maximum amount of chia seeds that can be included in bread products (5% whole seeds), breakfast cereals (10% whole seeds), baked goods (10% whole seeds), fruit and seed mixtures (10% whole seeds), fruit spreads (1% whole seeds), yogurts (1.3 g whole seeds/100 g yogurt or 4.3 g/330 g yogurt), fruit and vegetable juices and drinks (15 g/d whole or ground seeds), and ready-to-eat products (5% whole seeds) [29].

Due to their hydrophilic properties, chia seeds are used as an alternative to eggs and fat [112,113,114,115]. The seeds provide a characteristic mucilage texture to food and can absorb water up to 12 times their own weight [64]. Chia seed mucilage can stabilize emulsions; however, this property is influenced by the composition of the emulsion, and compared to other hydrocolloids, such as acacia gum, modified starch, and cellulose, chia seed mucilage has a low emulsifying activity index. Moreover, mucilage obtained from chia seeds is a rich source of polysaccharides, mainly cellulose, and hence can be used in the production of edible films and coatings [2,23].

Due to the fact, that chia seeds are a good source of omega-3 fatty acids (and have a favorable ratio of omega-3 to omega-6 fatty acids), they are commonly used by people on plant-based diets such as vegetarians or vegans [116]. In addition, due to the absence of gluten, chia seeds are a valuable ingredient for people with celiac disease to increase the nutritional value of the diet and for people on a gluten-free diet. Furthermore, wheat flour is substituted by chia seed skim flour as it contains more protein, dietary fiber, and bioactive compounds. In a study by Martinez et al., the nutritional and sensory properties of wheat flour biscuits were compared with biscuits containing skimmed chia, sesame, linseed, and poppy seeds. The authors found that biscuits baked using flour from various types of skimmed seeds contained twice as much protein as those made of wheat flour. Moreover, the content of dietary fiber and protein was the highest in cookies prepared using chia seed flour [48,63].

Chia seeds have also been used in meat processing, as an additive to sausages, for improving their nutritional and technological value. A study assessed the effect of *S. hispanica* seeds on lipid oxidation and proved that the addition of 0.5–1% chia seeds resulted in a reduction of fat oxidation in the meat product. This was attributed to the presence of numerous polyphenolic compounds, characterized by antioxidant activity, in chia seeds [33,117].

## 8. Plant Biotechnology Studies of *S. hispanica*

A review of the scientific literature shows that there are very few scientific papers on in vitro cultures studies of *S. hispanica*. These studies mainly concern the elaboration of micropropagation protocols—a technique used for rapid multiplication in vitro. Their short descriptions to date are briefly described below.

The aim of the study conducted by Carvalho et al. was to produce micropropagated *S. hispanica* embryos on two types of liquid medium. The researchers created Murashige–Skoog (MS) liquid medium, which differed in the presence of sucrose. After disinfecting the seeds, they were placed in glass vials with MS liquid medium with sucrose (30 g/L), (and without sucrose) and with plant growth regulators (PGRs). Cultures were grown at 25 ± 2 °C in a photoperiod of 16/8 h. Higher germination was observed in cultures grown on sucrose-containing media compared to media without sucrose. It was found that seedlings at day 14 had the highest shoot and root growth [118].

Zayova et al. conducted also a study on micropropagation of *S. hispanica*. High percentage of seed germination (100%) was recorded on MS medium enriched with 0.4 mg/L gibberellic acid (GA_3_) and 10 mg/L ascorbic acid after one week of culture. The maximum number of shoots per explant was obtained in cultures conducted on MS medium with 2 mg/L 6-benzyladenine (BA) after five weeks of culture. The best rooting of plants was achieved on MS medium with 0.1 mg/L indolyl-3-butyric acid (IBA) after four weeks of culture. Many plants were successfully adapted to ex vitro conditions with 95% survival rate [119].

Yadav et al. developed another micropropagation protocol for *S. hispanica*. *S. hispanica* seeds were germinated aseptically on ½ MS medium. Nodal explants obtained from in vitro germinated seedlings were maintained on MS medium with BA (1–5 mg/L) or kinetin (KIN) (1–5 mg/L) individually or with the addition of 1-naphthaleneacetic acid (NAA) (0.1–1 mg/L) and indolyl-3-acetic acid (IAA) (0.1–1 mg/L). The highest number of shoots per explant (9.02 ± 2.65) was found on culture medium containing 3 mg/L BA, which was also optimal for growth of regenerated shoots. Rooting was obtained on ½ MS medium with 1 mg/L IBA. The rooted shoots were acclimatized and transferred to natural conditions with 75% survival rate [120].

Marconi et al. initiated and optimized culture conditions for shoot and callus cultures of *S. hispanica*. Stem fragments were the best explant source to initiate in vitro cultures. Both used PGRs, IAA (0.57, 2.85, 5.70 μmol/L) and NAA (0.54, 2.70, 5.40 μmol/L), with or without BA (0.50 μmol/L) or KIN (0.46 μmol/L), induced adventitious root formation, both in the dark and in the light. In the dark, 2,4-dichlorophenoxyacetic acid (2,4-d) stimulated the development of *S. hispanica* embryogenic tissues, while in the light it promoted the initiation and formation of green and fast-growing callus, which lost their fragility over time. However, callus cultured on MS medium with NAA as PGR, maintained vitality for two years. Fatty acids were determined in the obtained callus cultures, but their content (0.73 g/100 g fresh weight) was much lower than in the seeds of parent plants (30.22 g/100 g fresh weight) [121].

Bueno et al. evaluated the in vitro behavior of different *S. hispanica* explants as material for the initiation of in vitro cultures. The explants tested were: young leaves, cotyledons, and stem fragments with two lateral buds. Explants were inoculated on MS culture medium with 0.1 µM of NAA; 0.1 µM of GA_3_; and 0, 0.5, 0.75, or 1 µM of BA. Inoculation of both leaves and cotyledons was unsuccessful. The good results of in vitro shoot survival were obtained for stem fragments. Furthermore, the greater plant proliferation, with up to 6.63 shoots per bud, was observed for BA in concentrations 0.75 and 1 µM. Higher concentrations of BA increased in vitro shoot formation [122].

## 9. Conclusions

*Salvia hispanica* L. is a plant species that has been used since antiquity and is now highly valued for its unique nutritional and potential medicinal properties. Chia seeds have recently become one of the most popular food ingredients, with a number of beneficial effects on the functioning of the human body. The data presented under our review indicate that they are also a valuable health-promoting dietary supplement as well as cosmetic ingredient. Scientific studies on pharmacological activities of chia seeds proved their potential valuable role in the prevention of diseases which currently are considered a global health problem. The research confirmed the cardioprotective, antihypertensive, antidiabetic, antiatherosclerotic, nephroprotective, anti-inflammatory, as well as antioxidant properties.

In recent years, chia seeds have been gaining popularity in food, diet supplement, and cosmetic industries, not only due to their valuable chemical composition and biological activity, but also due to their availability. Although *S. hispanica* occurs in very few natural habitats (Central America), it is successfully cultivated in many parts of the world. The popularity of *S. hispanica* is evidenced by (so far few) research in the field of plant biotechnology, the aim of which is to develop effective protocols for micro-propagation of this species under in vitro conditions.

Currently, chia seeds and products containing *S. hispanica* seed or herb extract or oil are widely available in health food stores, pharmacies, cosmetic stores, hypermarkets, and online stores.

The properties of *S. hispanica* species thanks to the seeds, undoubtedly merit special attention and interest. Due to their wide range of action and therapeutic value, chia seeds may be more widely used in the future, and *Salviae hispanicae semen* can be possibly included in the pharmacopoeial monographs of the European Union and other countries.

## Figures and Tables

**Table 1:** Content of the main EFAs (g/100 g dry seeds) in the *S. hispanica* seeds depending on their origin.

Unsaturated Fatty Acids			Origin of Chia Seeds	References
α-Linolenic Acid (ALA) n-3	Linoleic Acid (LA) n-6	Oleic Acid n-9		
(C_18_H_30_O_2_)	(C_18_H_32_O_2_)	(C_18_H_34_O_2_)		
61.9	19.9	7.5	West Mexico (Sinaloa)	[63]
63.4	19.8	8.2	Central West Mexico (Jalisco)	[63]
62.02	17.36	10.55	Southeast Brazil (Jacareia)	[62]
64.6	18.6	6.8	Argentina	[64]
61.92	18.99	6.77	France	[39]
60.94	19.16	6.87	France	[39]

**Table 2:** Content (mg/100 g dry seed) of phenolic acids in *S. hispanica* seeds—a review of studies.

Chlorogenic Acid	Ferulic Acid	Gallic Acid	Caffeic Acid	*p*-Coumaric Acid	Rosmarinic Acid	References
nd	t	1.15	2.74	nd	92.67	[72]
nd	3.587	4.256	12.536	2.596	65.398	[73]
4.59–10.20	nd	nd	0.30–0.68	nd	nd	[66]
nd	nd	0.005	-	0.024	nd	[74]
0.468	nd	nd	3.089	nd	nd	[62]
nt	nt	nd	nt	nd	nt	[75]

t—trace amounts; nd—not detected; nt—not tested.

**Table 3:** Content of individual macroelements and microelements (mg/100 g dry seeds) in the *S. hispanica* seeds.

Macroelements								References
Phosphorus (P)	Calcium (Ca)	Potassium (K)	Magnesium (Mg)	Sodium (Na)	Iron (Fe)	Manganese (Mn)	Sulfur (S)	
860	631	407	335	16	7.72	2.72	nt	[47]
530–640	430–480	550–620	330–350	140–150	7.69–9.39	2.48–4.05	150–200	[76]
696–799	580–624	666–870	369–403	nt	10.9–24.4	nt	nt	[77]
901.25–1247.60	561.50–806.00	nt	322.00–462.40	nt	11.73–14.27	nt	nt	[78]
919	456	726	449	0.26	9.18	3.79	nt	[74]
**Microelements**								
**Zinc (Zn)**		**Copper (Cu)**		**Molybdenum (Mo)**		**Selenium (Se)**		
4.58		0.92		0.20		0.06		[47]
3.65–3.76		0.63–1.32		nt		nt		[76]
6.0–6.9		nt		nt		0.08		[77]
0.60–10.00		1.87–2.42		nt		nt		[78]
6.47		1.86		nt		nt		[74]

nt—not tested.

**Table 4:** Content of individual vitamins in the *S. hispanica* seeds.

Vitamins	Content (mg per 100 g of dry seeds)	
	acc. to USDA (National Nutrient Database for Standard Reference) [71]	acc. to Knez et al. [9]
Niacin (vitamin B3)	8.80 mg	8.83 mg
Ascorbic acid (vitamin C)	1.60 mg	1.60 mg
Thiamine (vitamin B1)	0.60 mg	0.62 mg
Vitamin E	0.50 mg	0.50 mg
Riboflavin (vitamin B2)	0.20 mg	0.17 mg
Vitamin A	54 µg	54 µg

**Table 5:** Directions of biological activity of different *S. hispanica* seed raw materials.

Profile of Action	Raw Material	Mechanism	References
Lipid-lowering, hypoglycemic	Chia seed oil, chia seeds	Prevention of metabolic diseases by lowering TGs, TC, LDL, and VLDL and increasing HDL (by inhibiting 3-hydroxy-3-methylglutaryl coenzyme A (HMG-CoA) reductase), blocking the mevalonate metabolic pathway, lowering ALT and ASP, improving liver function, and improving postprandial glucose levels	[33,34,66,93]
Hypotensive	Chia seeds, chia seed flour, chia seed oil	Prevention of arterial hypertension by lowering systolic blood pressure	[82,84,96,97]
Weight-reducing	Chia seeds	Preventing overweight and obesity by inhibiting adipogenesis and reducing the level of PPAR-γ protein	[34,85]
Improving the function of the digestive tract	Chia seeds	Prevention of diseases of the gastrointestinal tract and intestinal dysbiosis by improving the absorption of nutrients (especially Fe and Zn), intensifying fermentation processes in the intestines, and increasing the production of short-chain fatty acids by intestinal bacteria, surface of intestinal villi, proliferation of enterocytes, and increase of beneficial intestinal bacteria, mainly *Bifidobacterium* and *Lactobacillus* in the cecum	[58]
Neuroprotective and anti-inflammatory	Chia seed oil	Prevention of neurodegenerative diseases by protective effect on microglial cells (HMC3) and reducing levels of inflammatory mediators (TNF-α, IL-6, NO, and H_2_O_2_) and ROS.	[33,92]
Preventing kidney stones	Chia seed methanol extract	Prevention of kidney stones by lowering the levels of creatinine, urea, and uric acid in the blood serum, inhibition of the initial stages of CaOx crystal formation, including nucleation, aggregation, and growth phases	[36,93,94,95]
Hepatoprotective	Chia seeds	Preventing liver disease by reducing the amount of intrahepatic fat, lipid deposition in hepatocytes, and increasing in plasma activity of antioxidant enzymes: catalase (CAT), glutathione peroxidase (GPx), and glutathione (GSH)	[67,90]
Antioxidant	Chia seeds	Preventing inflammation by reducing the level of CRP and inflammatory markers (tumor necrosis factor-α, nitric oxide, hydrogen peroxide, interleukin-6), antiradical activity, and the ability to inhibit lipoxygenase-5 and cyclooxygenases 1 and 2	[47,68,69]

**Table 6:** Raw materials obtained from *S. hispanica* registered in the CosIng database along with their uses [30].

Name according to CosIng	Application
*S. hispanica* seed	Abrasive, scrubbing agent
*S. hispanica* seed powder	Abrasive, scrubbing agent
*S. hispanica* seed oil	Moisturizing agent, nourishing effect on the epidermis
*S. hispanica* seed extract	Emollient, nourishing effect on the epidermis
*S. hispanica* herb oil	For the production of perfumes and aromas, functional fragrances
*S. hispanica* herb extract	For the production of perfumes and aromas, functional fragrances

## Supplementary Materials

The following are available online Figure S1. Chemical structures of the main fatty acids present in *S. hispanica* seeds; Figure S2. Chemical structures of the main amino acids present in *S. hispanica* seeds; Figure S3. Chemical structure of the main phenolic acids present in *S. hispanica* seeds; Figure S4. Chemical structures of the main flavonoids and epicatechin present in *S. hispanica* seeds.
