# Supplementary material for: The Current State of Knowledge on Salvia hispanica and Salviae hispanicae semen (Chia Seeds)
Source: Molecules. 2022 Feb 11;27(4):1207. doi: 10.3390/molecules27041207 (PMC8877361; doi:10.3390/molecules27041207)
Supplement: Supplementary file 1 [file molecules-27-01207-s001.zip › molecules-1560696-supplementary.pdf]

Supplementary material

The Current State of Knowledge on *Salvia hispanica* and *Salviae hispanicae semen* (Chia Seeds)

Sara Motyka [1], Katarzyna Koc [1], Halina Ekiert [1], Eliza Blicharska [2,*], Katarzyna Czarnek [3] and Agnieszka Szopa [1,*]

[1] Chair and Department of Pharmaceutical Botany, Jagiellonian University Medical College, ul. Medyczna 9, 30-688 Kraków, Poland; sara.motyka@doctoral.uj.edu.pl (S.M.); koc.kat.17@gmail.com (K.K.); halina.ekiert@uj.edu.pl (H.E.)

[2] Department of Analytical Chemistry, Medical University of Lublin, Chodźki 4a St., 20-093 Lublin, Poland

[3] Institute of Health Sciences, Faculty of Science and Health Sciences in Lublin, The John Paul II Catholic University of Lublin, ul. Konstantynów 1 H, 20-708 Lublin, Poland; kczarnek@kul.pl

* Correspondence: elizablicharska@umlub.pl (E.B.); a.szopa@uj.edu.pl (A.S.); Tel.: +48-814487182 (E.B.); +48-126205430 (A.S.)

Fatty acid	Type of fatty acid	Chemical structure
**Polyunsaturated fatty acids (PUFA)**		
α-Linolenic acid	Omega - 3	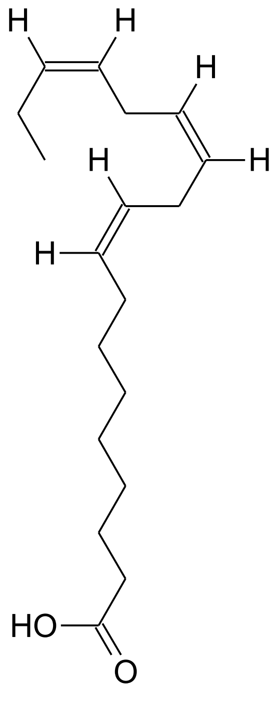
Linoleic acid	Omega - 6	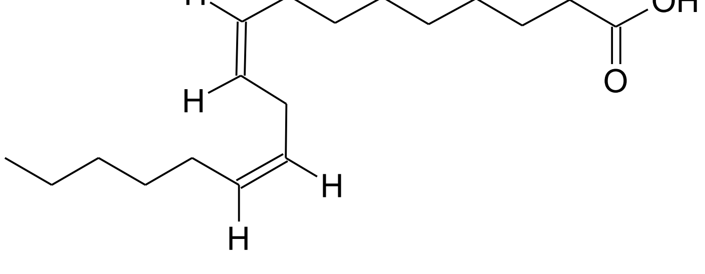
**Monounsaturated fatty acids (MUFA)**		

Oleic acid Omega - 9

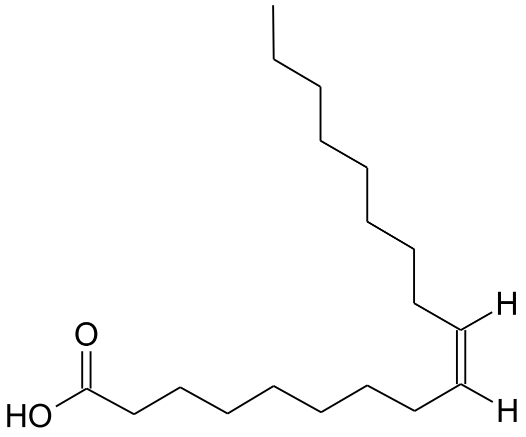

Saturated fatty acids (SFA)

Palmitic acid

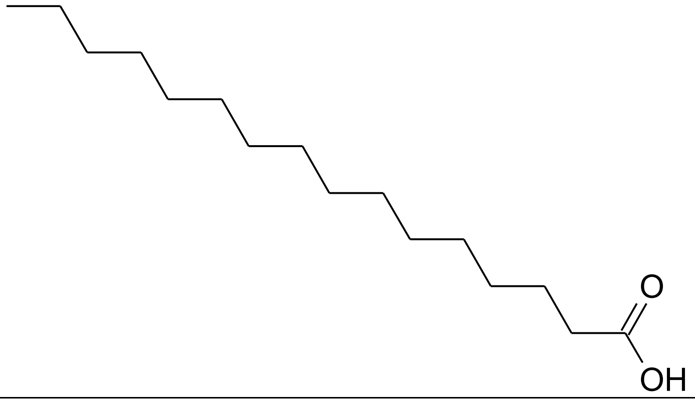

Stearic acid

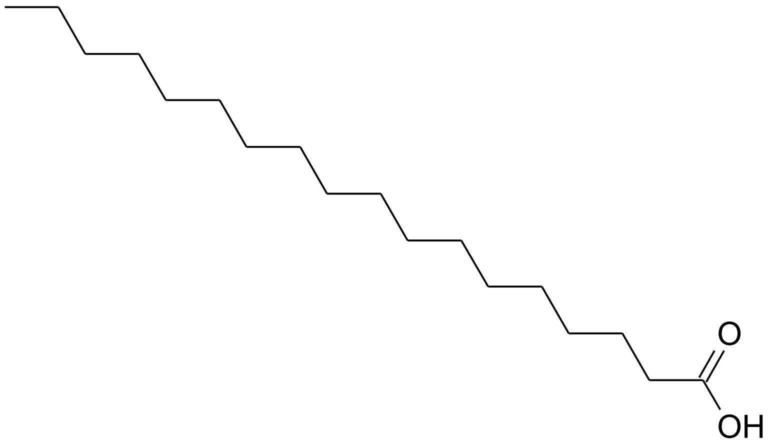

Figure S1. Chemical structures of the main fatty acids present in *S. hispanica* seeds.

Amino acid	Chemical structure
Glu	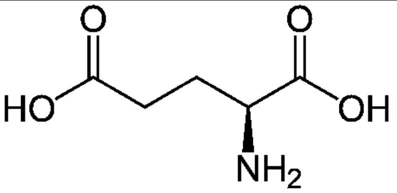
Arg	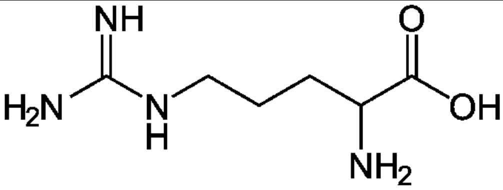
Asp	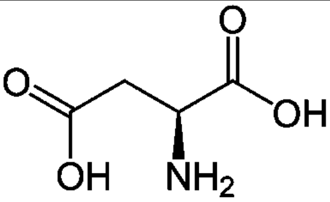
Leu	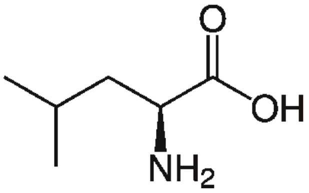
Ser	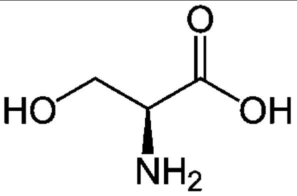
Ala	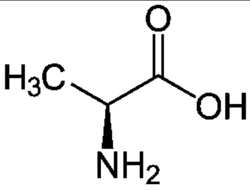
Phe	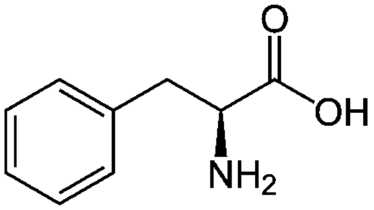

Figure S2. Chemical structures of the main amino acids present in *S. hispanica* seeds.

	Phenolic acid	Chemical structure
**Simple phenolic acids**	Gallic acid	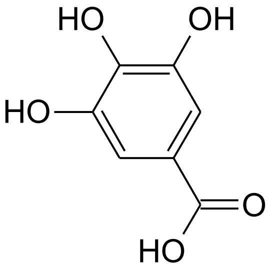
	Caffeic acid	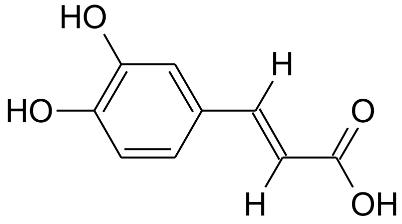
	Ferulic acid	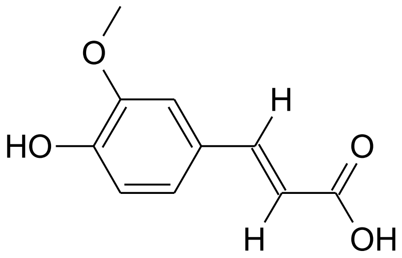
	p-Coumaric acid	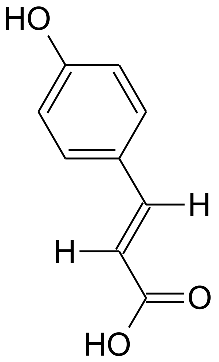
**Depsides**	Chlorogenic acid (3-O-cavoylquinic acid)	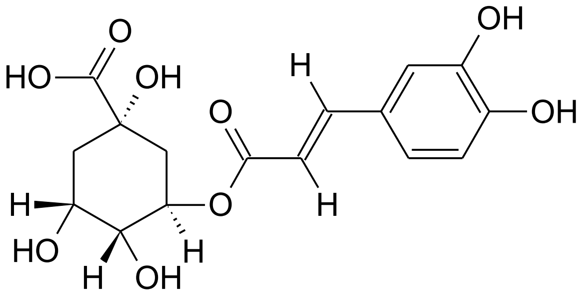

	Rosmarinic acid (ester of α-hydroxy-dihydrocaffeic acid and caffeic acid)	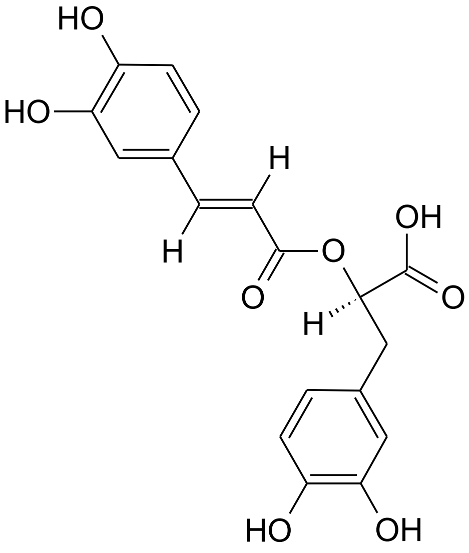

Figure S3. Chemical structure of the main phenolic acids present in *S. hispanica* seeds.

Compound		Chemical structure
**Flavonoid aglycones**	Apigenin	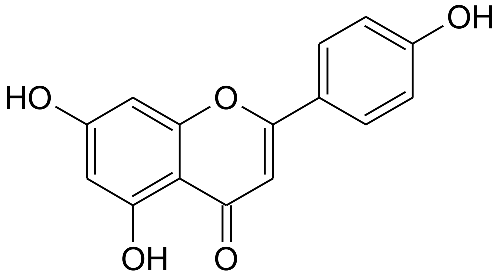
	Kaempferol	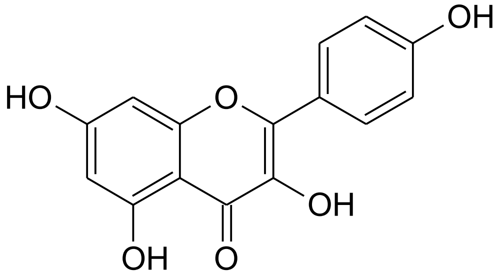
	Quercitin	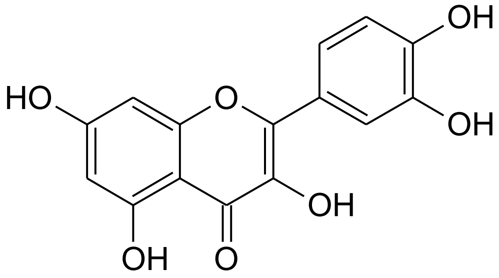
	Myricetin	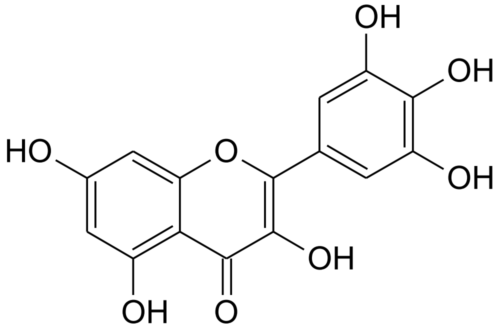

**Flavonoid glycosides**	Rutoside	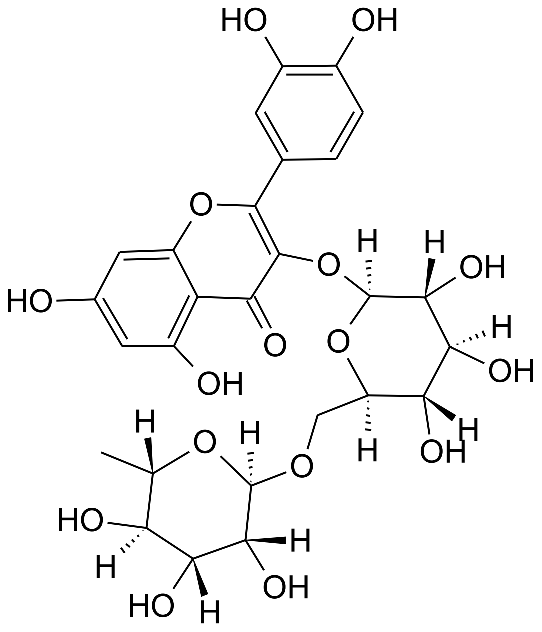
**Isoflavones**	Daidzenin	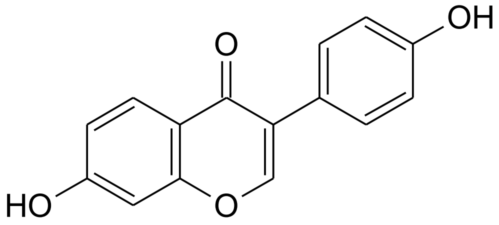
	Glycitin	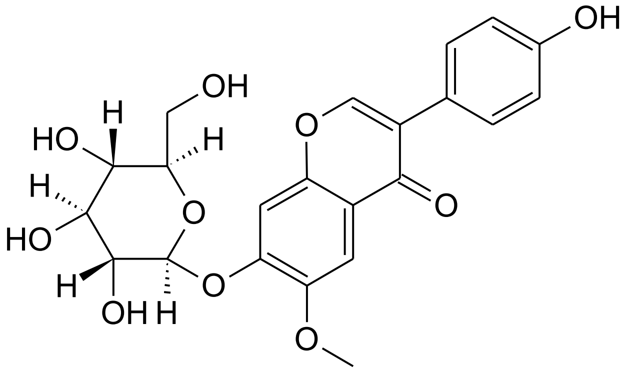
	Genistein	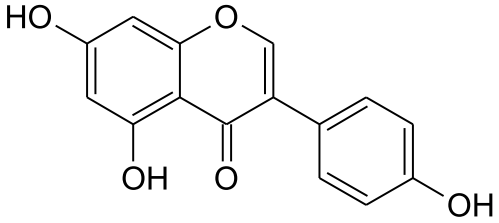

	Genistin	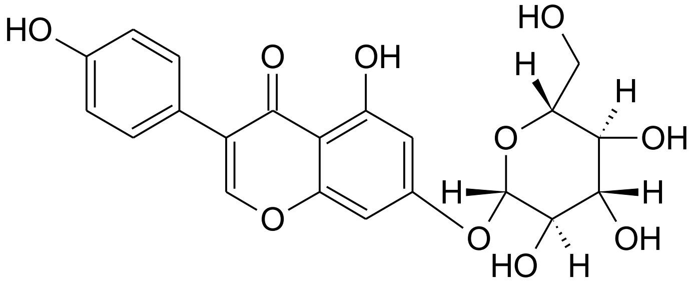
**Catechin derivative**	Epicatechin	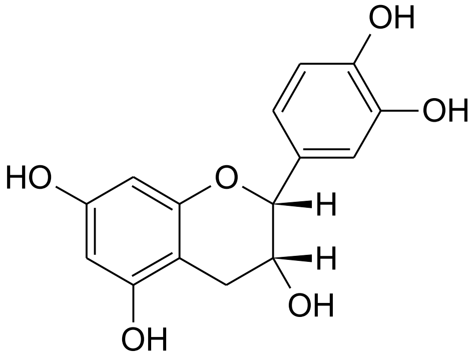

Figure S4. Chemical structures of the main flavonoids and epicatechin present in *S. hispanica* seeds.
